# Lifelong choline supplementation ameliorates Alzheimer’s disease pathology and associated cognitive deficits by attenuating microglia activation

**DOI:** 10.1111/acel.13037

**Published:** 2019-09-27

**Authors:** Ramon Velazquez, Eric Ferreira, Sara Knowles, Chaya Fux, Alexis Rodin, Wendy Winslow, Salvatore Oddo

**Affiliations:** ^1^ Arizona State University‐Banner Neurodegenerative Disease Research Center at the Biodesign Institute Arizona State University Tempe AZ USA; ^2^ School of Life Sciences Arizona State University Tempe AZ USA

**Keywords:** alpha7 nicotinic acetylcholine receptor, Alzheimer's disease, APP/PS1 mice, Aβ, choline supplementation, microglia activation, Sigma‐1 receptor, spatial memory

## Abstract

Currently, there are no effective therapies to ameliorate the pathological progression of Alzheimer's disease (AD). Evidence suggests that environmental factors may contribute to AD. Notably, dietary nutrients are suggested to play a key role in mediating mechanisms associated with brain function. Choline is a B‐like vitamin nutrient found in common foods that is important in various cell functions. It serves as a methyl donor and as a precursor for production of cell membranes. Choline is also the precursor for acetylcholine, a neurotransmitter which activates the alpha7 nicotinic acetylcholine receptor (α7nAchR), and also acts as an agonist for the Sigma‐1 R (σ1R). These receptors regulate CNS immune response, and their dysregulation contributes to AD pathogenesis. Here, we tested whether dietary choline supplementation throughout life reduces AD‐like pathology and rescues memory deficits in the APP/PS1 mouse model of AD. We exposed female APP/PS1 and NonTg mice to either a control choline (1.1 g/kg choline chloride) or a choline‐supplemented diet (5.0 g/kg choline chloride) from 2.5 to 10 months of age. Mice were tested in the Morris water maze to assess spatial memory followed by neuropathological evaluation. Lifelong choline supplementation significantly reduced amyloid‐β plaque load and improved spatial memory in APP/PS1 mice. Mechanistically, these changes were linked to a decrease of the amyloidogenic processing of APP, reductions in disease‐associated microglial activation, and a downregulation of the α7nAch and σ1 receptors. Our results demonstrate that lifelong choline supplementation produces profound benefits and suggest that simply modifying diet throughout life may reduce AD pathology.

## 1 INTRODUCTION

Alzheimer's disease (AD), the most prevalent neurodegenerative disorder worldwide, is clinically characterized by impairments in cognition, memory, and intellectual disabilities (LaFerla & Oddo, 2005). The neuropathological hallmarks of the AD brain are extracellular plaques composed predominately of the amyloid‐β (Aβ) peptide, intraneuronal tangles of hyperphosphorylated tau, and synaptic and neuronal loss (LaFerla & Oddo, 2005). Additionally, brain inflammation plays a significant role in neurodegeneration (Akiyama et al., 2000; Heppner, Ransohoff, & Becher, 2015; Holmes, Cunningham, Zotova, Culliford, & Perry, 2011; Schwab, Klegeris, & McGeer, 2010). Over the next few decades, there will be a dramatic increase in the prevalence of AD due to the advancing age of the global population; to this end, it is estimated that by the year 2050, there will be 14 million affected by this disorder in the United States alone (Alzheimer's Association, 2019). This is alarming given that no effective treatment options are available to prevent, treat, or manage AD.

Good nutrition is essential for proper cognitive function (Hu et al., 2013; Martinez Garcia, Jimenez Ortega, Lopez Sobaler, & Ortega, 2018). In particular, B‐like vitamins play important roles in mechanisms associated with attention, learning, and memory (Martinez Garcia et al., 2018). Choline is an essential nutrient that produces acetylcholine, a neurotransmitter essential for brain and nervous system functions including memory, muscle control, and mood (Jiang, West, & Caudill, 2014). Notably, acetylcholine activates alpha7 nicotinic acetylcholine receptors (α7nAchR) within microglia (Pohanka, 2012; Shytle et al., 2004). Microglia, the brain's resident immune cells, perform housekeeping functions that are essential to neuronal health (Tremblay et al., 2011). However, persistently activated microglia, which are increased in AD, contribute to a chronic state of brain inflammation leading to neuronal death (Itagaki, McGeer, Akiyama, Zhu, & Selkoe, 1989; Jin, Fang, Zhao, & Liu, 2015; Schwab et al., 2010; Zotova et al., 2011). Activated microglia are increased in the APP/PS1 mouse model of AD, and their presence is associated with an upregulation of the α7nAchR (Matsumura et al., 2015). Reductions in acetylcholine have been shown in the brains of patients with AD (Francis, 2005). Therefore, increasing acetylcholine may activate α7nAchR, which could decrease activated microglia. Furthermore, recent reports demonstrate that choline can also serve as an agonist for the Sigma‐1 receptor (σ1R), which plays a critical role in modulating brain inflammation (Brailoiu et al., 2019; Jin et al., 2015). Similar to α7nAchRs, σ1Rs are expressed in microglia, and dysregulation of these receptors can activate microglia and promote the release of proinflammatory molecules (Hall, Herrera, Ajmo, Cuevas, & Pennypacker, 2009). In fact, in vitro experiments have shown that σ1R agonists decrease microglia activation (Hall et al., 2009). Thus, increasing σ1R receptor function may reduce brain inflammation and neurodegeneration in AD.

Additional dietary choline is a putative treatment option that may prevent AD progression. Various reports have shown that supplementing the maternal (during gestation and lactation) diet with additional choline produces benefits in cognition and reduces neuropathology in a variety of neurodegenerative afflictions (Ash et al., 2014; Jiang et al., 2014; Kelly et al., 2016; Velazquez et al., 2019). While reports have shown minimal, if any, benefits of a choline supplementation during advanced stages of AD (Leermakers et al., 2015), it has yet to be determined whether choline supplementation throughout life may serve as a preventative therapy. Thus, there is a need to develop lifelong supplementation studies to evaluate the benefits of choline on AD pathology (Leermakers et al., 2015). The goal of the current study was to determine whether a lifelong regimen of choline supplementation (Ch+) reduces AD pathology and associated spatial reference memory deficits in female APP/PS1 mice.

## 2 RESULTS

**Figure 1:**
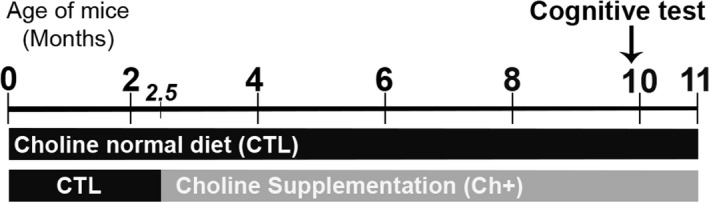
Experimental design. Starting at 2.5 months of age, female APP/PS1 and NonTg mice (APP/PS1 CTL = 10, NonTg CTL = 7, APP/PS1 Ch+ = 10, NonTg Ch+ = 7) were assigned to receive one of two concentrations of choline in diet; a diet containing 1.1 g/kg of choline (CTL) or a choline supplemented diet containing 5.0 g/kg of choline (Ch+). All mice were aged to 10 months and behaviorally tested in the hippocampal dependent Morris water maze task. Mice were subsequently sacrificed, and their brains were prepared for neuropathological assays

Starting at 2.5 months of age, we exposed female NonTg and APP/PS1 mice to either a control choline diet (CTL; 1.1 g/kg choline chloride) or a choline supplemented diet (Ch+; 5.0 g/kg choline chloride) containing 4.5 times the amount of choline consumed in the CTL group. All mice were aged to 10 months of age (APP/PS1 CTL = 10, NonTg CTL = 7, APP/PS1 Ch+ = 10, NonTg Ch+ = 7; Figure 1) and subsequently tested in the hippocampal‐dependent Morris water maze (MWM) task. After behavioral testing, all mice were sacrificed and had their brains extracted for neuropathological assessment.

### 2.1 Lifelong choline supplementation ameliorates spatial memory deficits in APP/PS1 mice

**Figure 2:**
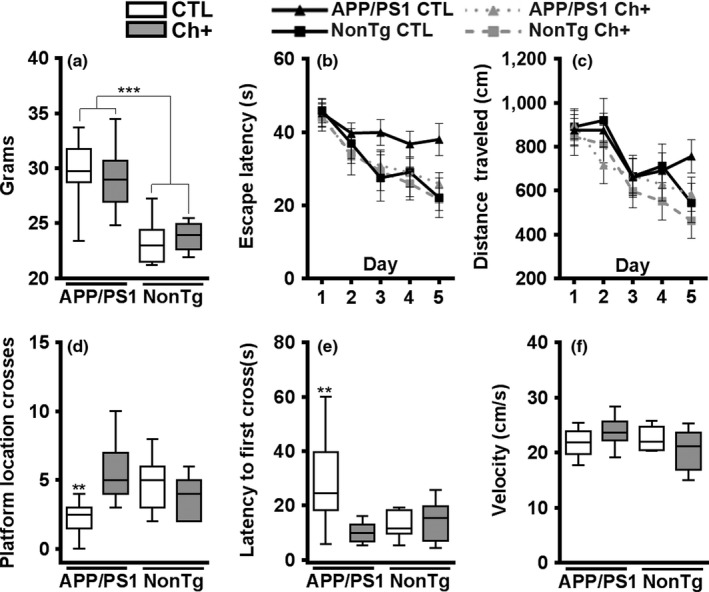
Lifelong choline supplementation significantly reduces spatial reference memory deficits. (a) Body weight analysis revealed that APP/PS1 mice are significantly heavier than NonTg (*p* < .0001), with no effect of diet (APP/PS1 CTL = 10, NonTg CTL = 7, APP/PS1 Ch+ = 10, NonTg Ch+ = 7). (b‐c) Escape latency and distance traveled to the platform during the learning phases of the Morris water maze (MWM). (d‐e) During the probe trial, we find that the APP/PS1 Ch+ mice cross the platform location significantly more times (*p* < .01) and first cross the platform location significantly faster (*p* < .01) than APP/PS1 mice on the CTL diet. (f) No differences in swim speed were detected among the four groups. Data are presented as box plots. The center line represents the median value, the limits represent the 25th and 75th percentile, and the whiskers represent the minimum and maximum value of the distribution. **p < .01, ***p < .001

We first assessed body weight of 10‐month‐old mice (APP/PS1 CTL = 10, NonTg CTL = 7, APP/PS1 Ch+ = 10, NonTg Ch+ = 7) and found a significant main effect of genotype (*F*_(1,30)_ = 46.900, *p* < .0001), where the APP/PS1 mice (*M* = 29.41 ± 0.893 g) weighed significantly more than the NonTg mice (*M* = 23.56 ± 0.646 g; Figure 2a). We then tested all mice on the Morris water maze (MWM) task. During the first 5 days, mice received four trials per day to locate a hidden platform using extra‐maze cues. We found a main effect of day for escape latency (*F*_(4,120)_ = 16.023, *p* < .001; Figure 2b) and distance traveled (*F*_(4,120)_ = 16.383, *p* < .001; Figure 2c), indicating learning throughout the 5 days of testing. On Day 6, the platform was removed, and the mice were given a single 60‐s probe trial. We found that the Ch+ groups had a significantly higher number of platform location crosses than their CTL counterparts (*F*_(1,30)_ = 4.415, *p* < .05, Figure 2d). Additionally, we found a significant genotype by diet interaction for platform crosses (*F*_(1,30)_ = 10.642, *p* < .01; Figure 2d). Bonferroni post hoc analysis show that the APP/PS1 Ch+ mice cross the platform location significantly more than the APP/PS1 CTL mice (*p* < .01) and performed as well as the NonTg mice. We also found a significant main effect of diet for latency to first cross the platform location, indicating that the Ch+ groups cross the platform location faster than the CTL mice (*F*_(1,30)_ = 5.219, *p* < .05, Figure 2e). Consistent with the number of platform location crosses, we found a significant genotype by diet interaction for latency to first cross the platform location (*F*_(1,30)_ = 6.630, *p* < .05; Figure 2e). Post hoc analysis reveals that APP/PS1 Ch+ mice take significantly less time to first cross the platform location than their CTL counterparts (*p* < .01) and performed as well as the NonTg mice. Notably, we found no significant differences in swim speed on Day 6 of testing (Figure 2f). These results clearly illustrate that lifelong Ch+ in APP/PS1 mice rescues spatial reference memory. Additionally, the diet effects suggest that choline supplementation was also beneficial for NonTg mice.

### 2.2 Lifelong choline supplementation reduces Aβ pathology in APP/PS1 mice

**Figure 3:**
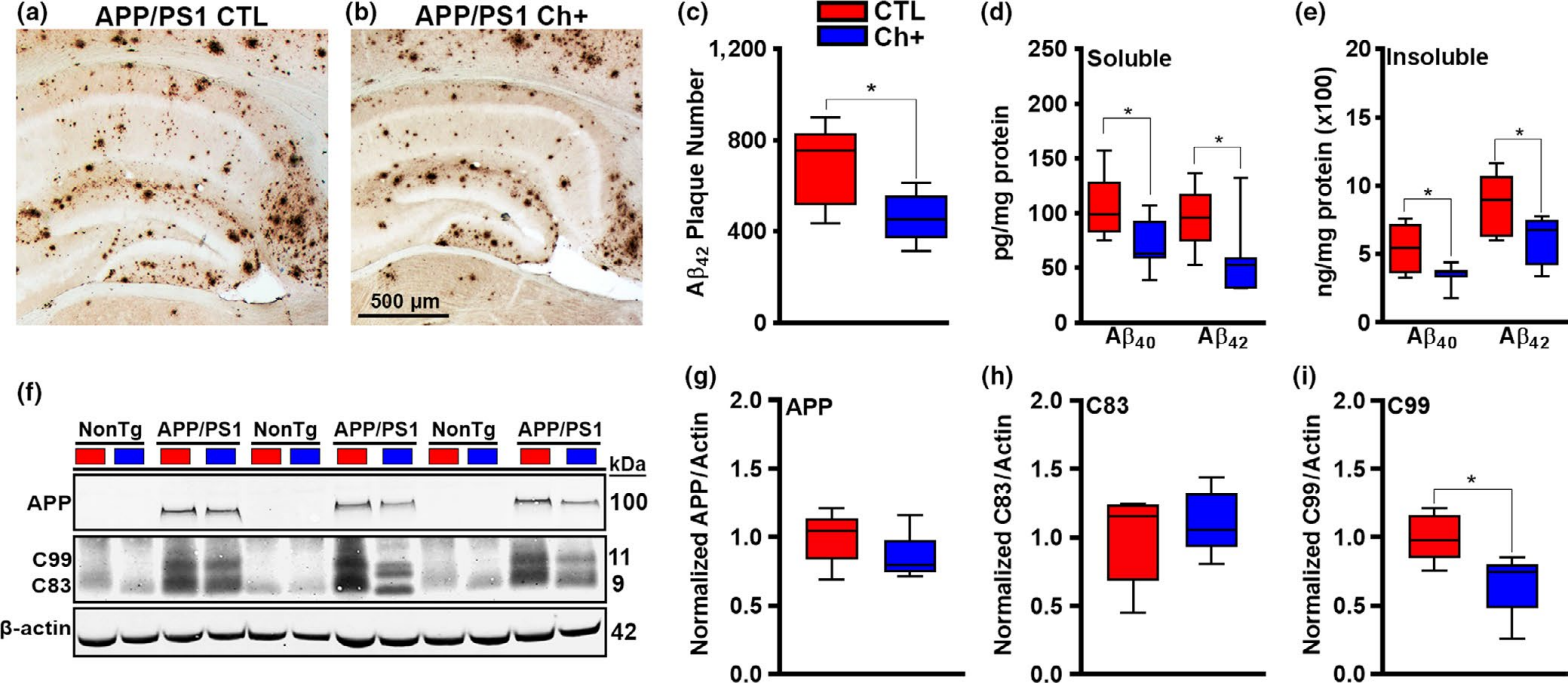
Lifelong choline supplementation significantly reduces Aβ pathology. (a‐b) Photomicrographs of Aβ_42_ plaques within the hippocampus of APP/PS1 CTL and Ch+ mice (*n* = 6 mice/group). (c‐e) Quantitative analysis reveals that lifelong Ch+ significantly reduces the number of Aβ_42_ plaques within the hippocampus of APP/PS1 mice (*p* < .05). Both soluble and insoluble forms of Aβ_40_ and Aβ_42_ were reduced in APP/PS1 Ch+ mice (*n* = 7 mice/group). (f‐i) Representative Western blot of APP processing. Quantitative analysis reveals that APP/PS1 Ch+ mice have significantly decreased levels of C99 compared to APP/PS1 mice on the CTL diet (*n* = 5 mice/group), illustrating a reduction in amyloidogenic processing of APP (*p* < .05). Data are presented as box plots. The center line represents the median value, the limits represent the 25th and 75th percentile, and the whiskers represent the minimum and maximum value of the distribution. *p < .05

At the end of the MWM testing, we sacrificed mice and prepared their brains for neuropathological and biochemical assessment. A key feature of AD is the accumulation of extracellular Aβ plaques (Laferla & Oddo, 2005). The Aβ peptide ranges from 36 to 43 amino acids in length, where Aβ_40_ and Aβ_42_ are the most abundant Aβ species. Aβ_42_ is more prone to aggregation and toxicity than Aβ_40_. Quantitative analysis of the Aβ_42_ plaque load (*n* = 6 mice/group) indicated a significant decrease of plaque number in the hippocampus of APP/PS1 Ch+ mice compared with APP/PS1 CTL mice (*t*_(10)_ = 2.875, *p* < .05, Figure 3a‐c). We next measured Aβ levels by sandwich ELISA (*n* = 7 mice/group) and found that both soluble Aβ_40_ and Aβ_42_ levels were significantly lower in APP/PS1 Ch+ mice compared with APP/PS1 CTL mice (soluble Aβ_40_: *t*_(12)_ = 2.576, *p* < .05; Aβ_42_: *t*_(12)_ = 2.328, *p* < .05; Figure 3d). Furthermore, insoluble levels of both Aβ_40_ and Aβ_42_ were significantly lower in APP/PS1 Ch+ mice compared to APP/PS1 CTL mice (insoluble Aβ_40_: *t*_(12)_ = 2.836, *p* < .05; A β42: *t*_(12)_ = 2.460, *p* < .05; Figure 3e). These results suggest that a lifelong regimen of Ch+ is sufficient to reduce the levels of toxic Aβ within the hippocampus, which could explain benefits in spatial cognition.

### 2.3 Lifelong choline supplementation ameliorates Aβ pathology by reducing the amyloidogenic processing of APP

To probe for a mechanism behind the Aβ changes after lifelong choline supplementation, we measured the steady state levels of full‐length APP and its two major C‐terminal fragments, C99 and C83 in APP/PS1 mice (*n* = 5 mice/group); Figure 3f). We found an equal level of APP (*t*_(8)_ = 1.305, *p* > .05; Figure 3f,g) and C83 (*t*_(8)_ = 0.606, *p* > .05; Figure 3f,h) between the CTL‐ and Ch+‐treated APP/PS1 mice. However, we found that Ch+ led to a significant decrease in the levels of C99 for the APP/PS1 mice (*t*_(8)_ = 2.597, *p* < .05; Figure 3f,i), which illustrates that lifelong Ch+ alters APP processing. Collectively, these results demonstrate a reduction in Aβ burden and a rescue of spatial memory deficits with lifelong Ch+.

### 2.4 Lifelong choline supplementation reduces the expression of activated microglia

Microglia housekeeping functions are essential to brain health. In contrast, overactivation of microglia, which occurs in AD, causes brain inflammation and can eventually lead to neuronal death (Heppner et al., 2015; Tremblay et al., 2011). To determine whether lifelong choline supplementation can reduce the levels of activated microglia, we double‐stained tissue with an antibody against Iba1, a marker of microglia, and CD68, a lysosomal marker (*n* = 6 mice/group) (Belfiore et al., 2018; Velazquez et al., 2019). CD68 and Iba1 colocalization signals can be used to determine the ratio of activated to total microglia.

**Figure 4:**
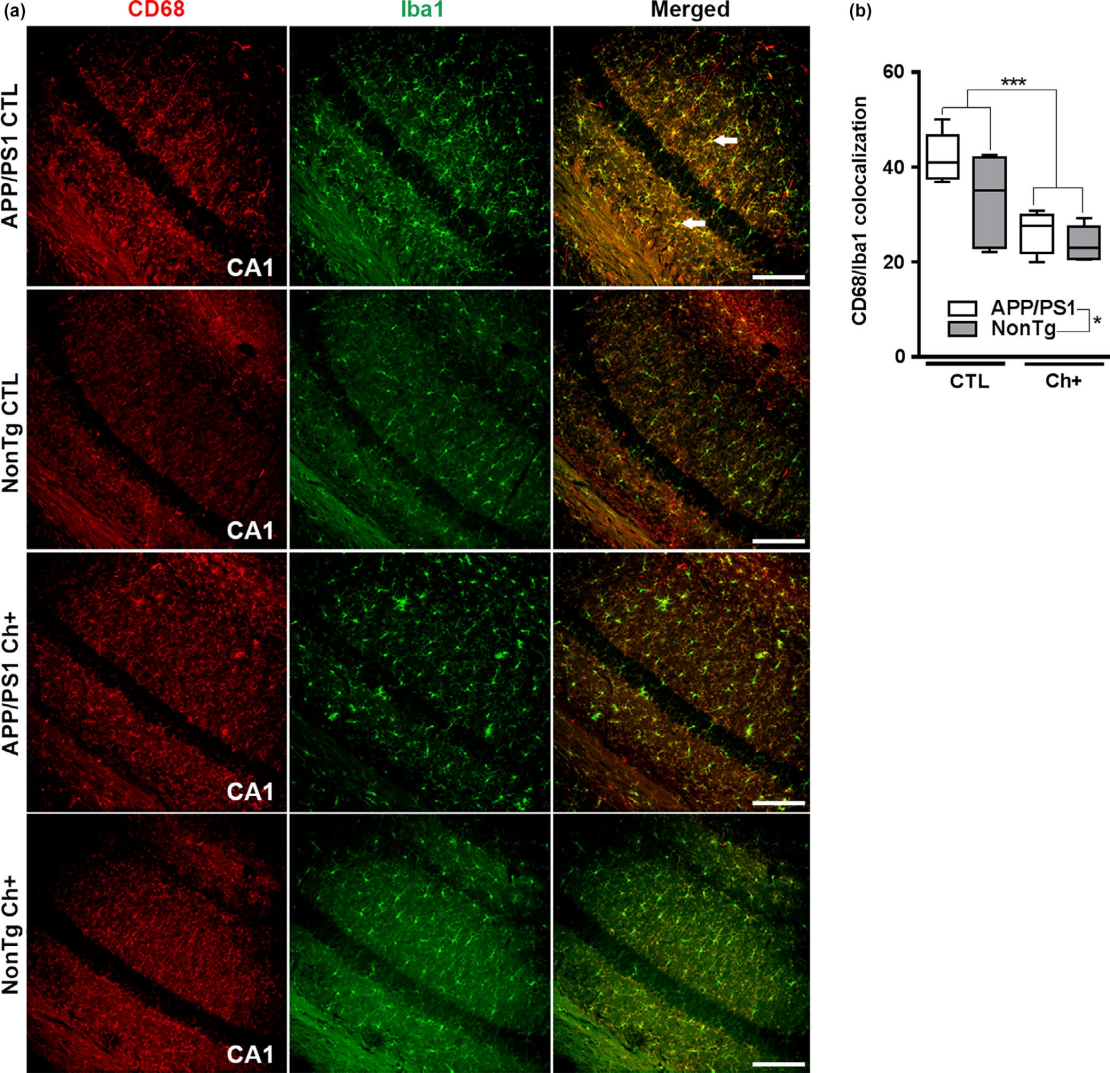
Lifelong choline supplementation reduces activated microglia. (a) Photomicrographs depicting the Cornus Ammonis 1 (CA1) of the hippocampus from APP/PS1 and NonTg mice fluorescently stained for CD68 and Iba1. Images taken at 10X; Scale bar = 150 µm (*n* = 6 mice/group). Arrows illustrate colocalization of CD68/Iba1. (b) Quantitative analysis reveals a significant main effect of genotype, where the APP/PS1 mice have a significantly higher intensity of yellow pixels of CD68/Iba1 colocalization than the NonTg mice (*p* < .05). Additionally, we find a significant main effect of diet, where the Ch+ groups show a significant reduction in CD68/Iba1 colocalization than the CTL groups (*p* < .001). Data are presented as box plots. The center line represents the median value, the limits represent the 25th and 75th percentile, and the whiskers represent the minimum and maximum value of the distribution. *p < .05, ***p < .001

We found a significant main effect of genotype, where the APP/PS1 mice had a significantly higher intensity of yellow pixels, indicating CD68/Iba1 colocalization, than the NonTg mice (*F*_(1,20)_ = 5.620, *p* < .05, Figure 4a,b). Additionally, we found a significant main effect of diet, where the Ch+ groups showed significantly less CD68/Iba1 colocalization than the CTL groups (*F*_(1,20)_ = 28.455, *p* < .0001, Figure 4a,b). Collectively, these results show that lifelong Ch+ reduces the levels of activated microglia, thereby mitigating the detrimental effects of brain inflammation associated with AD‐like pathology.

### 2.5 Lifelong choline supplementation alters the expression of the alpha7 nicotinic acetylcholine receptor (α7nAchR)

**Figure 5:**
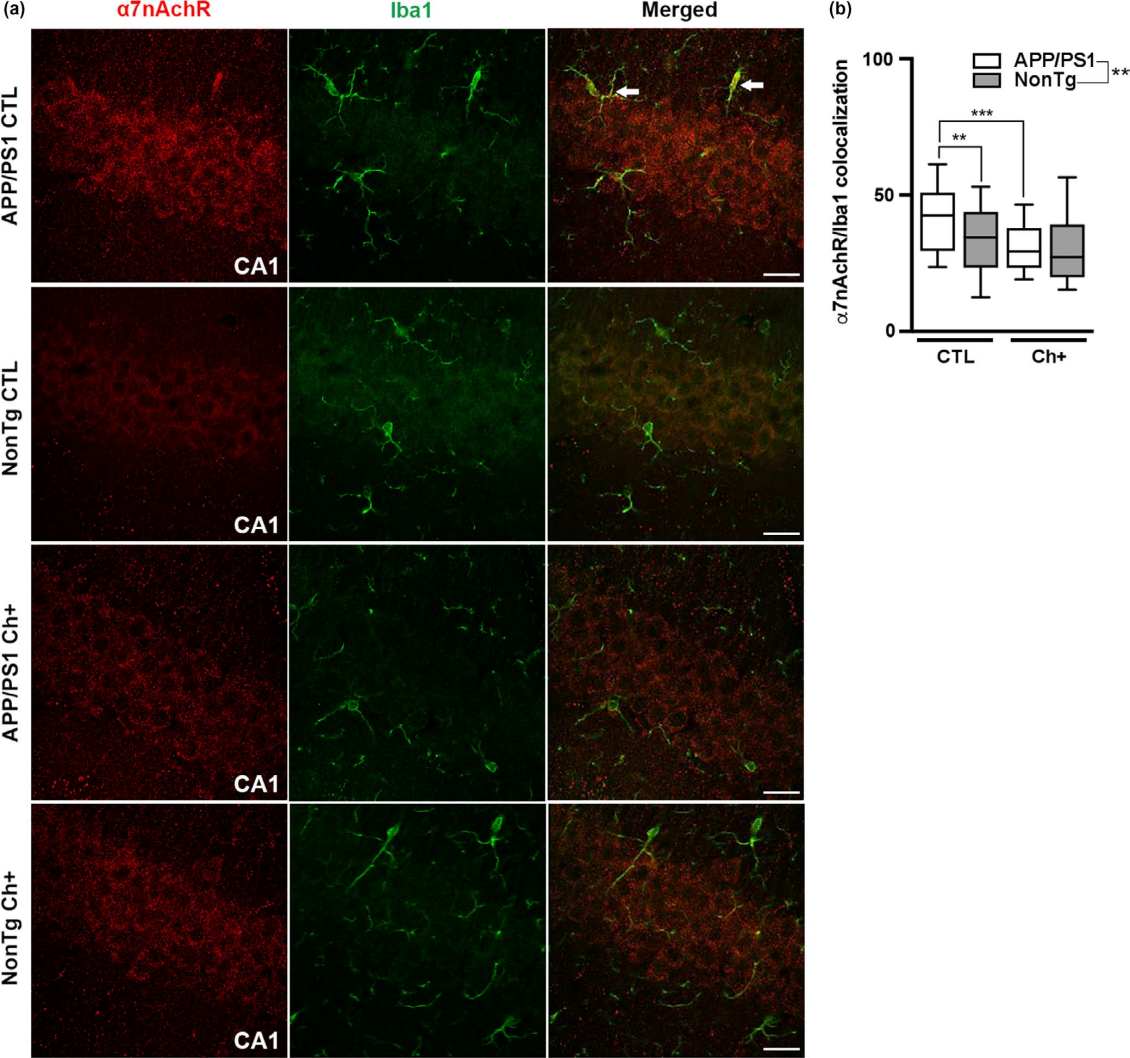
Lifelong choline supplementation alters the expression of the alpha7 nicotinic acetylcholine receptor (α7nAchR) within microglia. (a) Photomicrographs depicting the Cornus Ammonis 1 (CA1) of the hippocampus from APP/PS1 and NonTg mice fluorescently stained for α7nAchR and Iba1. Images taken at 40X; scale bar = 25 µm (*n* = 6 mice/group). (b) Quantitative analysis reveals a significant main effect of genotype, where the APP/PS1 mice have a significantly higher intensity of yellow pixels of α7nAchR/Iba1 colocalization than the NonTg mice (*p* < .01). Additionally, we find a significant main effect of diet, where the Ch+ groups show a significant reduction in α7nAchR/Iba1 colocalization than the CTL groups (*p* < .05). A genotype by diet interaction was found, where the APP/PS1 Ch+ mice show a significant reduction of α7nR/Iba1 colocalization compared to the CTL counterparts (*p* < .001). The center line represents the median value, the limits represent the 25th and 75th percentile, and the whiskers represent the minimum and maximum value of the distribution. **p < .01, ***p < .001

Increased expression of the α7nAchR is associated with activated microglia in APP/PS1 mice (Matsumura et al., 2015). α7nAchRs are activated by acetylcholine, and choline serves as a precursor of this neurotransmitter (Pohanka, 2012). To determine whether lifelong choline supplementation alters the expression of the α7nAchR in microglia, we double‐stained tissue with an antibody against α7nAchR and Iba1 (*n* = 6 mice/group). Quantitative analysis revealed a significant main effect of genotype, where the APP/PS1 mice had a significantly higher intensity of yellow pixels, indicating increased α7nAchR/Iba1 colocalization, than the NonTg mice (*F*_(1,20)_ = 15.00, *p* < .01, Figure 5a,b). Additionally, we found a significant main effect of diet, where the Ch+ groups showed significantly less α7nAchR/Iba1 colocalization than the CTL groups (*F*_(1,20)_ = 4.644, *p* < .05, Figure 5a,b). We also found a significant genotype by diet interaction (*F*_(1,20)_ = 3.992, *p* < .05, Figure 5a,b). Post hoc analysis indicated that the APP/PS1 CTL mice have a significantly increased colocalization of α7nAchR/Iba1 than the NonTg CTL mice (*p* < .01). Additionally, we found a significant decrease in intensity of α7nAchR/Iba1 colocalization in APP/PS1 Ch+ mice compared to the CTL counterparts (*p* < .001). These results suggest that lifelong Ch+ reduces the expression of the α7nAchR within microglia, which may be associated with decreased microglia activation.

### 2.6 Lifelong choline supplementation alters the expression of the Sigma‐1 receptor (σ1R)

**Figure 6:**
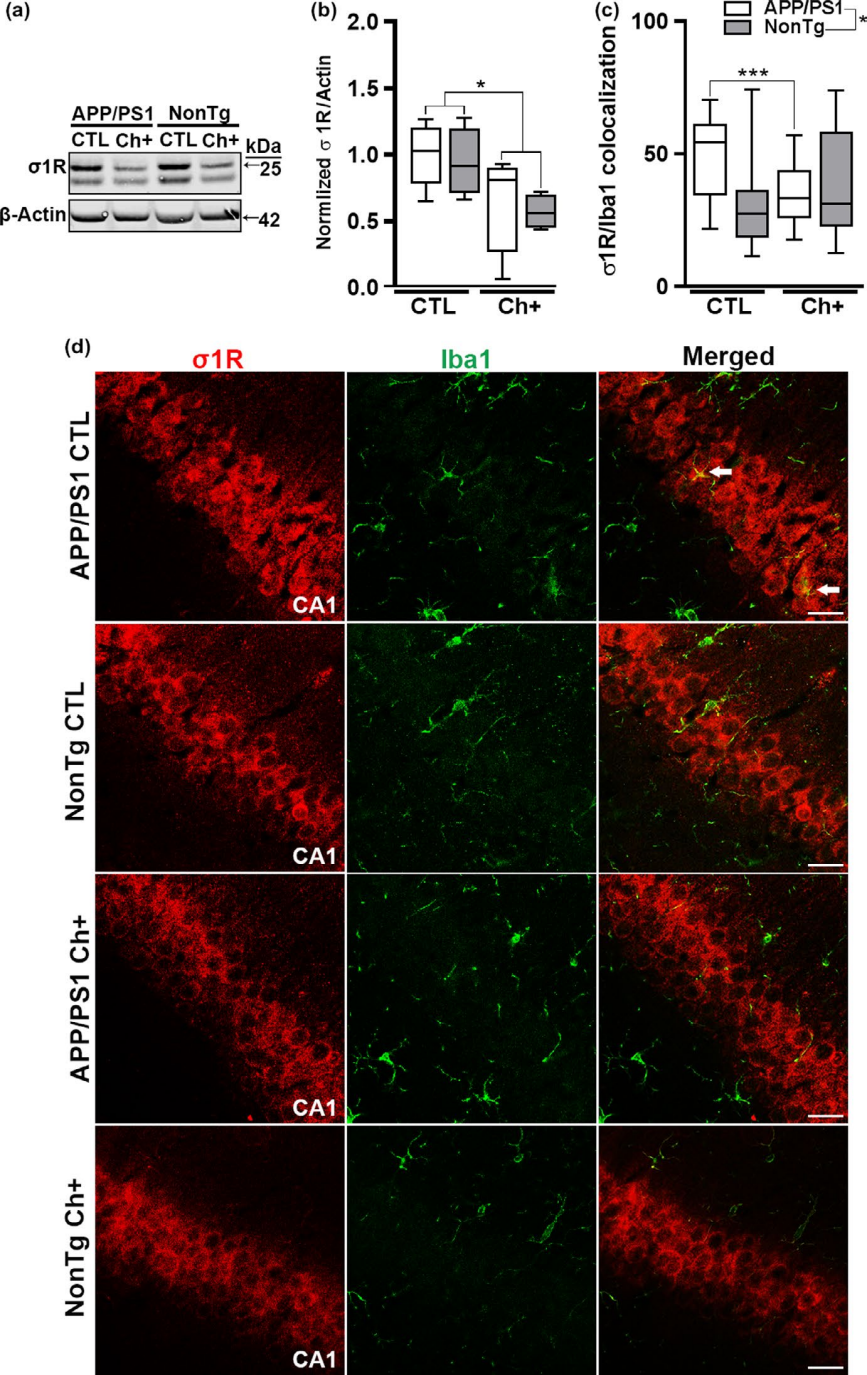
Lifelong choline supplementation alters the expression of the Sigma‐1 receptor (σ1R) within microglia. (a‐b) Representative Western blot of σ1R levels. Quantitative analysis of σ1R protein levels reveals a significant reduction with Ch+ (*p* < .05; *n* = 5/APP group; *n* = 4/NonTg group). (c‐d) Quantitative analysis reveals a significant main effect of genotype, where the APP/PS1 mice have a significantly higher intensity of yellow pixels of σ1R/Iba1 colocalization than the NonTg mice (*p* < .05, *n* = 6 APP/PS1 CTL, *n* = 5 APP/PS1 Ch+, *n* = 6 NonTg CTL, *n* = 6 NonTg Ch+). Additionally, we find a significant genotype by diet interaction where the APP/PS1 Ch+ mice show a significant reduction in σ1R/Iba1 colocalization than the APP/PS1 CTL mice (*p* < .001). Photomicrographs depicting the Cornus Ammonis 1 (CA1) of the hippocampus from APP/PS1 and NonTg mice fluorescently stained for the σ1R and Iba1; images taken at 40×; scale bar = 25 µm. Data are presented as box plots. The center line represents the median value, the limits represent the 25th and 75th percentile, and the whiskers represent the minimum and maximum value of the distribution. *p < .05, ***p < .001

The σ1R modulates brain inflammation (Francardo et al., 2014; Jin et al., 2015; Mancuso et al., 2012). The σ1R is a chaperone protein at the endoplasmic reticulum that modulates calcium signaling through the inositol 1,4,5‐trisphosphate receptor (IP3 receptor) (Brailoiu et al., 2019; Jin et al., 2015). σ1Rs are expressed on microglia, and dysregulation of these receptors can activate microglia and promote the release of proinflammatory molecules (Hall et al., 2009). Research suggests that microglia activation may be inhibited by σ1R agonists (Francardo et al., 2014; Jin et al., 2015; Mancuso et al., 2012). This is relevant as recent evidence shows that choline serves as a ligand for the activation of the σ1R (Brailoiu et al., 2019). Efficient activation of the σ1R may be one mechanism by which lifelong choline supplementation reduces inflammation. To determine whether lifelong choline supplementation alters σ1R expression, we measured its steady state levels via immunoblot (*n* = 5 mice/APP group; *n* = 4 mice/NonTg group). We found that lifelong choline supplementation significantly reduced the expression of the σ1R, independent of genotype (*F*_(1,14)_ = 8.617, *p* < .05; Figure 6a,b). To determine the cell specificity of the σ1R downregulation, we double‐stained tissue with an antibody against the σ1R and Iba1 (*n* = 6 APP/PS1 CTL, *n* = 5 APP/PS1 Ch+, *n* = 6 NonTg CTL, *n* = 6 NonTg Ch+). We found a significant main effect of genotype for yellow pixels, indicating an increase in σ1R/Iba1 colocalization in APP/PS1 mice compared to the NonTg mice (*F*_(1,19)_ = 6.331, *p* < .05, Figure 6c,d). Additionally, we found a significant genotype by diet interaction for σ1R/Iba1 colocalization (*F*_(1,19)_ = 12.135, *p* < .001). Post hoc analysis indicated that the APP/PS1 Ch+ mice have a significantly decreased colocalization of σ1R/Iba1 than the APP/PS1 CTL group (*p* < .0001, Figure 6c,d). Collectively, these results indicate that higher dietary choline reduces the expression of the σ1R within microglia cells in APP/PS1 mice, which in turn may decrease microglia activation.

## 3 DISCUSSION

Our results illustrate that a lifelong regimen of additional choline produces significant benefits on AD‐like pathology and associated cognitive deficits in female APP/PS1 mice. We find that Ch+ improves spatial reference memory and reduces Aβ burden. Additionally, we find that the levels of activated microglia in both APP/PS1 and NonTg mice are reduced with Ch+. This suggests that additional dietary choline may be an avenue to reduce brain inflammation in both neurodegenerative disease and the nondiseased aging brain (von Bernhardi, Tichauer, & Eugenin, 2010; Brown, 2009). Mechanistically, we find that a regimen of lifelong Ch+ significantly altered the levels of the α7nAch and σ1 receptors, which are involved in the regulation of microglia activation (Francardo et al., 2014; Hall et al., 2009; Jin et al., 2015; Mancuso et al., 2012; Matsumura et al., 2015; Pohanka, 2012). Collectively, these results highlight the important role of additional dietary choline throughout life and demonstrate the potential of Ch+ as a strategy for healthy brain aging.

Lifelong Ch+ significantly reduced Aβ burden in the hippocampus of female APP/PS1 mice. Notably, we found decreased C99 levels and a reduction of soluble and insoluble levels of Aβ_40_ and Aβ_42_, which indicates a reduction in amyloidogenic processing of APP. Previous reports have shown that increasing maternal choline intake substantially reduces Aβ burden in APP/PS1 mice (Mellott et al., 2017; Velazquez et al., 2019). Mechanistically, it was demonstrated that reductions in Aβ burden can be accomplished through the alteration of homocysteine (Hcy), a neurotoxic amino acid that binds to Aβ and facilitates it aggregation and accumulation (Velazquez et al., 2019). Notably, choline converts Hcy to methionine through the donation of methyl groups by choline's metabolite, betaine (Zeisel, 2017). Thus, it is tempting to speculate that the reduced Aβ levels in APP/PS1 mice exposed to lifelong Ch+ are linked to lower Hcy levels, which in turn reduce Aβ aggregation and accumulation. Future work will elucidate this association.

Our results show a reduction of activated microglia in the hippocampus of mice that received a lifelong regimen of Ch+. Activated microglia have been shown to be detrimental to neurons (Brown, 2009; Heppner et al., 2015; Itagaki et al., 1989). Chronic microglia activation is a feature of AD and is associated with the presence of Aβ plaques (von Bernhardi et al., 2010; Itagaki et al., 1989; Schwab et al., 2010; Zotova et al., 2011). Activated microglia release proinflammatory cytokines and other molecules that, when not properly regulated, are implicated in mediating neurodegeneration (Brown, 2009; Heppner et al., 2015; Itagaki et al., 1989). Consistent with the data reported here, previous work found that supplementation of choline in the maternal diet reduced the expression of activated microglia in aged APP/PS1 mice (Velazquez et al., 2019). It was not determined whether the additional maternal choline directly reduced microglia activation or indirectly by reducing the production of Aβ plaques (Velazquez et al., 2019).

A mechanism by which lifelong Ch+ may have reduced disease‐related microglia is through the activation of the α7nAch and σ1 receptors. Various lines of evidence have shown that microglia activation is associated with an increase expression of the α7nAchR (Matsumura et al., 2015; Shytle et al., 2004). Acetylcholine binds to α7nAchRs, thereby allowing for the activation of this receptor (Pohanka, 2012). Reductions in acetylcholine have been shown in the brains of patients with AD (Francis, 2005). Increasing acetylcholine production may increase activation of the α7nAchR, thereby downregulating this receptor. Our results show decreased expression of the α7nAchR within microglia and a decrease in activated microglia in APP/PS1 Ch+ mice. These findings suggest that increased expression of α7nAchR in APP/PS1 CTL mice could be due to less activation by acetylcholine. Therefore, supplementing with precursor choline may increase binding to α7nAchR receptor, thereby attenuating microglia activation. Additionally, σ1Rs are expressed in microglia and inflammatory responses can be inhibited by activation of the σ1R (Hall et al., 2009; Jin et al., 2015; Mancuso et al., 2012). Interestingly, recent work demonstrates that choline binds to the σ1R and serves as an agonist for its activation (Brailoiu et al., 2019). Our results show a decrease in the expression of the σ1R within microglia in APP/PS1 Ch+ mice, which is likely due to down‐regulation resulting from increased availability of choline. To this end, the increased expression of the σ1R in APP/PS1 CTL mice may be due to reduced sensitivity of the receptor (Nguyen et al., 2015). Given that choline serves as an agonist for σ1R, and since activation of this receptor is linked to a reduction in microglia activation, it is tempting to speculate that Ch+ facilitates the activation of σ1R, which results in its downregulation, thereby reducing activated microglia.

The current established adequate intake level of choline for adult (>19 years of age) women is 425 mg/day and 550 mg/day for adult men (“US Standing Committee on the Scientific Evaluation”, 1998). A study examining daily human choline intake on an ad libitum diet found averages to be lower than the recommended daily intake amount (RDI), especially in women (Fischer et al., 2007). This is interesting, given the higher incidence of AD seen in women compared to men (Alzheimer's Association, 2019). Additionally, a converging line of evidence indicates that even the current RDI may not be optimal for a proper aging process (Blusztajn, Slack, & Mellott, 2017; Caudill, Strupp, Muscalu, Nevins, & Canfield, 2018; Wallace et al., 2018). This suggests that additional choline in diet may be beneficial in preventing neuropathological changes associated with the aging brain. The tolerable upper limit (TUL) of choline unlikely to cause side effects for adult females and males (>19 years of age) is 3,500 mg/day, which is 8.24 times higher than the 425 mg/day recommendation for females and 6.36 times higher than the 550 mg/day recommendation for males (US Standing Committee on the Scientific Evaluation, 1998). Given that we supplemented with 4.5 times the RDI, we are well within the TUL of choline. Side effects when surpassing the TUL of choline include hypotension and fishy body odor (“US Standing Committee on the Scientific Evaluation”, 1998). Notably, choline can be found in various foods (U.S. Department of Agriculture, 2019). High levels of choline are found in chicken liver (3 oz; 247 mg), eggs (1 large egg with yolk;147 mg), beef grass‐fed steak (3 oz; 55 mg), wheat germ (1 oz toast; 51 mg), milk (8 oz; 38mg), and Brussel sprouts (1/2 cup; 32 mg; (Wallace et al., 2018)). Additionally, vitamin supplements containing choline, for example, choline bitartrate and choline chloride, are widely available at affordable costs (Wallace et al., 2018).

In conclusion, our results confirm that a diet regimen with additional choline throughout life can reduce the pathological burden and associated cognitive deficits seen in female APP/PS1 mice. Notably, we find that benefits of lifelong Ch+ are associated with a reduction in microglia activation, which is likely through the activation of the α7nAch and σ1 receptors, given choline's role as a precursor for acetylcholine and as an agonist for the σ1R. Remarkably, we also found Ch+ benefits on reduced microglia activation in NonTg mice, which demonstrates advantages for the aging brain. If these results generalize to humans, the adjustment to a choline‐supplemented diet throughout life may mitigate the estimated increased prevalence observed for AD. Given that we found beneficial effects of lifelong Ch+ in nondiseased aged mice, this type of intervention may be beneficial for the general population, thereby providing substantial benefits to offset age‐related pathologies.

## 4 MATERIALS AND METHODS

### 4.1 Animals and diet regimen

The generation of the APP/PS1 mice has been described previously (Jankowsky et al., 2004). The APP/PS1 mice are hemizygous for the amyloid precursor protein (APP) Swedish mutations (KM670/671Nl) and presenilin1 (PS1) deltaE9 mutation. We have backcrossed the APP/PS1 mice for 12 generations into a pure 129/SvJ background. All protocols were approved by the Institutional Animal Care and Use Committee of Arizona State University and conform to the National Institutes of Health Guide for the Care and Use of Laboratory Animals. All mice were group‐housed (4–5 mice/cage) to lessen the environmental impoverishment of the laboratory setting. We used female mice for this study. At 2.5 months of age, female APP/PS1 and wild‐type (NonTg) mice were randomly assigned to receive one of two concentrations of choline chloride in their diet (Harlan laboratories). The control diet (CTL; 1.1 g/kg choline chloride) contains the standard concentration of choline chloride found in rodent diets and is currently considered to provide the “normal daily recommendation” of choline intake (Ash et al., 2014; Meck & Williams, 2003; Velazquez et al., 2013). The choline intake in the choline supplemented diet (5.0 g/kg choline chloride; Ch+) is approximately 4.5 times the amount of choline consumed in the CTL group. This diet regimen is similar to that used in previous studies examining the benefits of gestational choline supplementation (Ash et al., 2014; Powers et al., 2017; Velazquez et al., 2013, 2019).

### 4.2 Morris water maze

The MWM task was conducted as previously described (Velazquez et al., 2018). To assess spatial learning, each animal was given four trials per day for a total of 5 days. The location of the hidden platform remained in the same quadrant for all the animals; however, the start location was pseudorandomly selected. Each animal was given 60 s to locate the hidden platform. If the animal failed to reach the hidden platform in the 60 s, they were gently guided to its location. On Day 6, the platform was removed, and animals were given a 60‐s probe trial to assess spatial reference memory.

### 4.3 Protein extraction and Western blots

Proteins were extracted and Western blots were performed under reducing conditions as we previously detailed (Velazquez et al., 2017). Quantitative analyses of the Western blots were obtained by normalizing the intensity of the protein of interest with its loading control, β‐actin. imagej software was used to quantify the intensity of the bands of interest.

### 4.4 Brain tissue processing and histology

For immunohistochemistry analysis, hemispheres were fixed in 4% paraformaldehyde for 48 hr. Tissue was then sectioned (50 μm thick) using a sliding vibratome and stored in PBS with 0.02% sodium azide. Immunohistochemistry was performed as we previously described (Velazquez, Shaw, Caccamo, & Oddo, 2016). To quantify Aβ_42_ pathology load, images from six mice/group were taken with a Zeiss Axio Imager A1 using a 5× objective. Images were photomerged to rebuild the image, and plaque number was obtained using imagej. The experimenter was blinded to the group allocation.

To evaluate microglia activation, we used immunofluorescent labeling. Specifically, we co‐ labeled brain sections with Iba1 and CD68 antibodies followed by secondary Alexa‐Fluor labeling, as previously described (Belfiore et al., 2018; Velazquez et al., 2019). Iba1 recognizes total microglia, while CD68 is a lysosomal marker that detects activated microglia. To evaluate the expression of receptors within microglia, we colabeled a separate set of brain sections with Iba1 and α7nAchR antibodies, and Iba1 and σ1R antibodies followed by secondary Alexa‐Fluor labeling. Immunofluorescent images were taken on a Leica confocal microscope using a 10× and 40× objectives with 1.5× zoom. We took five pictures of the hippocampus from one section per mouse and thus analyzed 30 images per group. Images were analyzed using imagej; colocalization data were obtained using the ImageJ “Coloc2” plug‐in.

### 4.5 Antibodies

All the antibodies used in this study have been validated by the manufacturer for use in mouse tissue. The following antibodies were purchased from Abcam: α7nAchR (Catalog #ab216485, dilution 1:200), CD68 (Catalog #ab955, dilution 1:200), and σ1R (Catalog #ab151288, dilution 1:1000); Cell signaling: β‐actin (Catalog #4967, dilution 1:1000); Millipore: Amyloid Precursor Protein, C‐terminal (Catalog #171610, dilution 1:3000), antiAβ42 (Catalog #5078P, dilution 1:200), and 6E10 (Catalog MAB1560, dilution 1:3000); Novus Biologicals: Goat‐Iba1 (Catalog #NB100‐2833, dilution 1:200); and Wako: Mouse‐Iba1 (Catalog NCNP24, dilution 1:200).

### 4.6 ELISA

To asses soluble and insoluble levels of Aβ, we used the Life Technologies ELISA Kit as previously described (Velazquez et al., 2016). Briefly, soluble or insoluble fractions of brain tissue homogenates were processed and read in a plate reader (BioTek) at 450 nm in precoated, flat‐bottom 96‐well plates according to the manufacturer's instructions. The range of Aβ detection was between 10 and 1,000 pg/ml. For each assay kit, cross‐reactivity with other species of Aβ, APP, or tau was negligible when concentrations were <10 mg/ml. The concentration of Aβ (picograms per milliliter per sample) present in the homogenate was the dependent variable used for statistical analysis.

### 4.7 Statistical analyses

Repeated‐measures ANOVAs were used to analyze the behavioral experiments followed by Bonferroni's corrected post hoc tests, when appropriate. Two‐way factorial ANOVAs were used to analyze body weight, Western blot quantifications, and probe trail‐dependent measures. Student's unpaired *t* tests were employed for comparison of APP/PS1 mice when appropriate. Outliers were defined as exhibiting a mean ≥ two standard deviations from the mean of the group. Examination of descriptive statistics revealed no violation of any assumptions that required the use of statistical test other than the ones used. Significance was set to *p* ≤ .05.

## CONFLICT OF INTEREST

None declared.

## AUTHOR CONTRIBUTIONS

R.V. designed and conducted experiments, analyzed the data, and wrote the manuscript. E.F. conducted the activated microglia experiments, analyzed the data, and edited the manuscript. S.K. conducted the immunoblots, analyzed the data, and edited the manuscript. C.F. acquired images and analyzed the data. A.R. conducted immunostaining and edited the manuscript. W.W. conducted immunostaining experiments and edited the manuscript. S.O. helped design the experiments and edited the manuscript.

## Data Availability

The data that support the findings of this study are available from the corresponding author, R.V., upon reasonable request.
